# *Lippia javanica* (Zumbani) herbal tea infusion attenuates allergic airway inflammation via inhibition of Th2 cell activation and suppression of oxidative stress

**DOI:** 10.1186/s12906-021-03361-8

**Published:** 2021-07-05

**Authors:** Mvuyisi O. M. Mfengu, Mathulo Shauli, Godwill A. Engwa, Hannibal T. Musarurwa, Constance R. Sewani-Rusike

**Affiliations:** 1 Department of Human Biology, Faculty of Health Sciences, Walter Sisulu University PBX1, 5117, Mthatha, South Africa; 2 Department of Biological and Environmental Sciences, Faculty of Natural Sciences, Walter Sisulu University PBX1, 5117, Mthatha, South Africa

**Keywords:** Asthma, Oxidative stress, Inflammation, Antioxidant, Th2-mediated cell immune response

## Abstract

**Background:**

*Lippia javanica* (lemon bush) is commonly used in the treatment of respiratory ailments, including asthma in southern African countries but there is no scientific evidence to support this claim. This study investigated the anti-inflammatory, antioxidant and anti-asthmatic effects of *L. javanica* using a rat model of asthma.

**Methods:**

A 5% w/v *L. javanica* tea infusion was prepared and characterised by liquid chromatography–mass spectrometer (LC-MS). Animals were intraperitoneally sensitized with ovalbumin (OVA) and subsequently challenged intranasal with OVA on day 15 except the control group. Animals were grouped (*n* = 5/group) for treatment: unsensitised control, sensitised control, sensitised + prednisolone and sensitised + *L. javanica* at 50 mg/kg/day and 100 mg/kg/day – equivalent to 1 and 2 cups of tea per day, respectively. After 2 weeks of treatment, bronchoalveolar lavage fluid (BALF) was collected for total and differential white blood cell (WBC) count. Nitric oxide (NO), lipid peroxidation and antioxidants were also assessed in BALF. Ovalbumin specific IgE antibody and inflammatory cytokines: IL-4, IL-5, IL-13 and TNF-alpha were measured in serum. Lung and muscle tissues were histological examined.

**Results:**

*L. javanica* was rich in phenolic compounds. OVA sensitisation resulted in development of allergic asthma in rats. *L. javanica* treatment resulted in a reduction in total WBC count as well as eosinophils, lymphocytes and neutrophils in BALF. *L. javanica* inhibited Th2-mediated immune response, which was evident by a decrease in serum IgE and inflammatory cytokines: IL-4, IL-5, IL-13 and TNF-α. *L. javanica* treatment also reduced malondialdehyde (MDA) and NO, and increased superoxide dismutase, glutathione and total antioxidant capacity. Histology showed significant attenuation of lung infiltration of inflammatory cells, alveolar thickening, and bronchiole smooth muscle thickening.

**Conclusion:**

*L. javanica* suppressed allergic airway inflammation by reducing Th2-mediated immune response and oxidative stress in OVA-sensitized rats which may be attributed to the presence of phenolic compound in the plant*.* This finding validates the traditional use of *L. javanica* in the treatment of respiratory disorders.

## Background

Chronic respiratory diseases such as chronic bronchitis, asthma and obstructive pulmonary disease are among the leading causes of death worldwide, with asthma rated the most common [1]. Asthma is an airway inflammatory disease characterised by variable expiratory airflow limitation associated with bronchoconstriction, increased mucus secretion and exaggerated bronchial hyper-responsiveness. The disease presents with difficulty in breathing and wheezing associated with airway remodelling as the disease progresses [2, 3]. Clinically, asthma can be allergic or non-allergic, distinguished by the presence or absence of IgE antibodies to common environmental allergens [4]. Globally, over 300 million people suffer from asthma, and based on current trends, it is estimated that this number will rise to over 400 million by 2025 [5, 6]. In Africa, it is estimated that 47.9 million people are asthmatic [7]. The prevalence of asthma in South Africans is between 6 and 10% with 20% of school-going age pupils between 9 and 15 years of age experiencing symptoms of asthma [8].

Asthma is a Type 1 allergic disease caused by immune responses to allergens which is mediated by Th2 cells [9]. When allergens are processed by dendritic cells and presented to T-helper lymphocytes (Th), proinflammatory cytokines including interleukins (IL)-4, IL-5, IL-13 and tumour necrosis factor alpha (TNF-α) are produced [10, 11]. IL-4 activates memory B cells to secrete immunoglobulins and induces class switching from IgM to IgE. IL-4 also promotes incorporation of IgE on mast cells resulting in allergic degranulation, exacerbates goblet cell secretions resulting in airway obstruction [12, 13]. IL-5 induces lung eosinophilia which is the critical response in the induction of airway hyper-responsiveness and allergic asthma. IL-13 supports IL-5 effects, increases inducible nitric oxide synthase (iNOS) and airway fibrosis and remodelling [14–17]. TNF-α is a chemo-attractant for eosinophils and neutrophils via such mediators as cysteinyl leukotrienes C_4_ and D_4_ [18]_._ Additively, these cytokines (IL-4, IL-5, IL-13 and TNF-α) result in increased inflammatory cells in lungs, reduced lung compliance and increased airway resistance [19, 20]. Ideally, invasive or non-invasive methods such as whole-body plethysmography, the forced oscillation technique, the interrupter technique, rhinomanometry and acoustic rhinometry have been used to assess airway resistance and lung function [21] in conjunction with biochemical parameters. However, studies in ovalbumin-induced asthma have consistently shown an association between lung dysfunction and biochemical markers of inflammation and oxidative stress [22–24].

Oxidative stress is known to aggravate airway inflammation by inducing diverse pro-inflammatory mediators, enhancing bronchial hyper-responsiveness, stimulating bronchospasm, and increasing mucus secretion [25]. It has been reported that elevated levels of reactive oxygen species (ROS) can trigger some intracellular signalling cascades such as the mitogen-activated protein kinase (MAPK) and nuclear factor-κB (NF-κB) that are potentially proinflammatory leading to the expression of inflammatory cytokines and chemokines [26]. Also, losing control of oxidants in the airway may supress immune tolerance and induce the initiation of Th2-dominant immunity which is an initial phase of development of airway allergic inflammation [27]. There are epidemiological evidences that suggest increased reactive oxygen species (ROS) is associated with asthma (Mak and Chan-Yeung, 2006; Bowler, 2004) [28, 29]. Endogenous antioxidants such as superoxide dismutase (SOD), catalase, glutathione peroxidase as well as glutathione (GSH) are the natural host defence system against oxidative stress that quench and eliminate ROS [27]. Studies have shown that there is deficiency of antioxidants in asthma and exogenous antioxidants such as vitamin C improve disease and symptom severity [30, 31].

Although there are several drugs on the market used to manage asthma such as β2 adrenergic agonists, anticholinergics, immunosuppressors and mast cell stabilizers [32, 33], these drugs have toxic side effects after long term use [32]. Herbal medicines for the treatment of a variety of ailments are accepted and tolerated by most communities in South Africa and elsewhere in Africa. *Lippia javanica* commonly known as Lemon bush (English), Zumbani tea (Shona) and Zinziniba (IsiXhosa) [33, 34] is an herb whose leaves are eaten as a vegetable, food additive, used to brew tea drunk for pleasure or as a home-remedy for respiratory ailments. Locally, the leaf infusion is used for treating respiratory ailments such as asthma, bronchitis, common cold, pneumonia, tuberculosis [35] and lately for combating respiratory effects associated with coronavirus disease (COVID-19) caused by the new Severe Acute Respiratory Syndrome coronavirus (SARS-CoV-2) [36]. *Lippia javanica* is rich in polyphenols [37], a class of phytochemical molecules which are well-known antioxidants and also reported to possess anti-allergic and anti-inflammatory properties [38]. We have previously reported *Lippia javanica* to possess higher in vitro antioxidant capacity compared to rooibos tea [39]. Despite the reported common use of *L. javanica* for asthma and other inflammation-associated respiratory ailments in South Africa, Zimbabwe and other central and southern African countries, to our knowledge, there is no scientific evidence to support its effectiveness in mitigating allergen induced airway inflammation. Therefore, the aim of this study was to investigate the anti-inflammatory, antioxidant and anti-asthmatic effects of *Lippia javanica* using a rat model of asthma.

## Materials and methods

### Plant collection

*Lippia javanica* tea leaves were purchased from a local specialty shop in Harare, Zimbabwe. The tea was sold as unprocessed leaves by the manufacturer (Utsanzi® Lippia herbal tea).

### Phenolic profiling by liquid chromatography- mass spectrometer (LC-MS)

The *L. javanica* dried leaf extract (2 g) was ground in a mortar with pestle and extracted with 50% methanol in deionised water containing 1% formic acid (15 mL) by soaking overnight and extracting in an ultrasonic bath (0.5 Hz, Integral Systems, RSA) for 60 min at room temperature. The extracts were centrifuged (Hermle Z160m, 3000 g for 5 min) and transferred to vials for LC-MS analysis. A Waters Synapt G2 Quadrupole time-of-flight (QTOF) mass spectrometer (MS) connected to a Waters Acquity ultra-performance liquid chromatography (UPLC) (Waters, Milford, MA, USA) was used for high-resolution UPLC-MS analysis. Electrospray ionization was used in negative mode with a cone voltage of 15 V, desolvation temperature of 275 °C, desolvation gas at 650 L/h, and the rest of the MS settings optimized for best resolution and sensitivity. Data were acquired by scanning from m/z 150 to 1500 m/z in resolution mode as well as in MSE mode. In MSE mode two channels of MS data were acquired, one at a low collision energy (4 V) and the second using a collision energy ramp (40–100 V) to obtain fragmentation data as well. Leucine enkephalin was used as lock mass (reference mass) for accurate mass determination and the instrument was calibrated with sodium formate. Separation was achieved on a Waters HSS T3, 2.1 × 100 mm, 1.7 μm column. An injection volume of 2 μL was used and the mobile phase consisted of 0.1% formic acid (solvent A) and acetonitrile containing 0.1% formic acid as solvent B. The gradient started at 100% solvent A for 1 min and changed to 28% solvent B over 22 min in a linear way. It then went to 40% B over 50s and a wash step of 1.5 min at 100% B, followed by re-equilibration to initial conditions for 4 min. The flow rate was 0.3 mL/min and the column temperature was maintained at 55 °C. Ion mobility data was obtained using the same UPLC gradient and column as above and IMS Wave velocity was set at 332 m/s and wave height at 20.2 V. Polyalanine was used for the calibration and calculations and the rest of the settings were according to Rautenbach et al. [40]. Sample was run in triplicate and results reported as μg/L.

### Preparation of *Lippia javanica* tea infusion for animal treatments

Two hundred millilitre (200 ml) of boiled deionised water was added to 10 g of the dry crushed *L. javanica* tea leaves followed by stirring with a magnetic stirrer for 10 min then steeping for 30 min and strained with a fine mesh tea strainer followed by vacuum filtration through Whatman No. 1 filter paper [41]. The resultant tea infusion filtrate was used for animal treatments.

### Experimental animals and ethics

This study was conducted in accordance with the ethical guidelines of Animal Care and Use and the Animal Research: Reporting of In Vivo Experiments (ARRIVE) guidelines [42]. Ethics clearance for this study was obtained from Faculty of Health Sciences Research Ethics Committee, Walter Sisulu University, South Africa with ethics clearance reference number 063/2018. Twenty-five female Wistar rats were purchased from South African Vaccine Producers, Johannesburg, South Africa. The rats were housed in the Department of Human Biology animal holding facility, Walter Sisulu University for one week to allow the animals to acclimatize to the new environment before the initiation of the study. The animals were kept in polypropylene cages with absorbent pine shaving bedding, having open steel tops and housed 5 rats/cage. The room was temperature controlled at 22 ± 2 °C on 12-h light: dark cycles. The rats had free access to water and rat pellet feed (Epol, South Africa: 180 g/kg protein, 120 g/kg moisture, 25 g/kg fat, 18 g/kg calcium, 7 g/kg phosphorus and 60 g/kg fibre). The bedding was changed and cages cleaned twice a week. The rats were taken care of adhering to standards stipulated in the South African National Standards [43].

### Animal ovalbumin sensitization and airway challenge

**Fig. 1:**
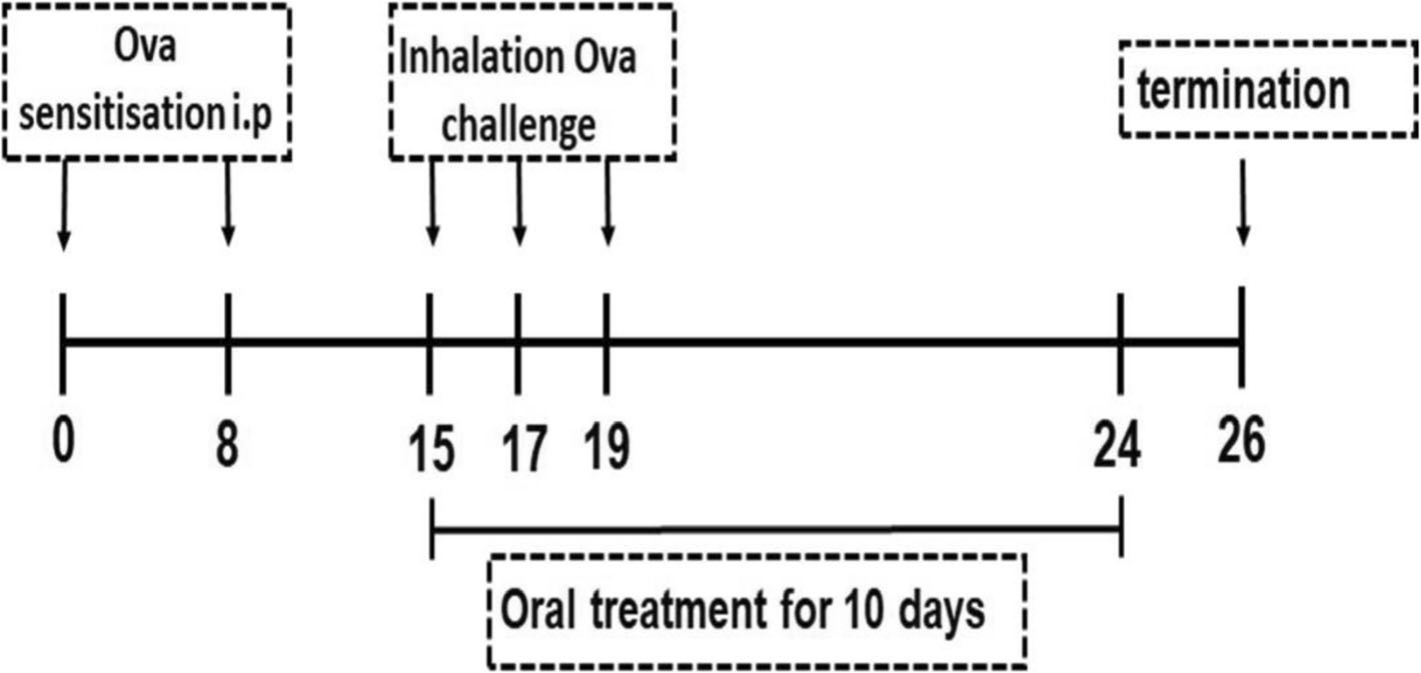
**Schematic diagram of the experimental protocol.** Diagram showing induction by intraperitoneal OVA injections (Day 0 and 8), aerosolized airway challenge (Days 15, 17 and 19), treatment period (Days 15 to 24) and termination (Day 26)

To induce airway inflammation, Wistar rats were immunized with 0.3 mL injections of 90 μg of ovalbumin (OVA) prepared from dried egg white mixed with 600 μg aluminium hydroxide adjuvant. Intraperitoneal (i.p.) injections were given 7 days apart (day 0 and day 8) as previously described [44]. The control group was injected with 0.3 ml distilled water. On day 15, a week after the booster OVA i.p. injection, the animals were challenged for 30 min with aerosolized 1% OVA in normal saline (nebulisation). The rats were placed in an enclosed chamber which was saturated with aerosolized OVA using a mist nebuliser (Healthease). The control group was nebulised with normal saline only. Nebulisation was repeated on days 17 and 19 (Fig. 1). Treatment was initiated on day 15, the day of first aerosolized OVA airway challenge.

### Treatment protocol

The female Wistar rats were divided into 5 treatment groups (*n* = 5/group) as follows:

1. Normal control (unsensitised, treated with water)
2. Sensitised untreated control (treated with water)
3. Sensitised, treated with 50 mg/kg body weight (bw) *L. javanica*
4. Sensitised, treated with 100 mg/kg bw *L. javanica*
5. Sensitised, treated with 10 mg/kg bw prednisolone (positive control)

Animals were treated daily by oral gavage. The 50 and 100 mg/kg bw doses for *L. javanica* are equivalent to 1 and 2 cups human dose equivalent respectively using the body surface area as a conversion factor of daily therapeutic dose from human to rat using a multiplication factor of 6.2 according to Reagan-Shaw et al. [45]. Treatment was given 30 min before each nebulisation and continued over 10 days until day 24. The rats were weighed weekly to adjust treatment according to the body weights. The study design is summarised in Fig. 1.

### Terminal procedures and sample collection

On day 26 of the study, the animals were euthanised by CO_2_ inhalation. Blood was collected by cardiac puncture into plain tubes, allowed to clot and centrifuged at 3000 rpm (Eppendorf 5810 R) to obtain serum for cytokine and IgE assays. Bronchoalveolar lavage fluid (BALF) was collected using 3 × 1 mL ice-cold phosphate buffered saline via trachea cannulation. The BALF was centrifuged at 3000 rpm at 4 °C for 10 min and the supernatant was stored at − 20 °C for antioxidant and nitric oxide (NO) determination. The pellet was used for inflammatory cell counts. A portion of the lung tissue was fixed in 10% neutral buffered formalin for histopathological analysis.

### Inflammatory cell counts in bronchoalveolar lavage fluid (BALF)

The resuspended BALF pellet was used for total white blood cell (WBC) count using a Neubauer haemocytometer. For differential inflammatory cell counts, slide smears of the pellet were prepared and stained with DiffQuick staining reagent (Thermo Fisher). Eosinophils, lymphocytes, macrophages and neutrophils were identified and counted using standard morphologic determinants and expressed as percent (%) [46].

### Biochemical analyses in bronchoalveolar lavage fluid (BALF)

Bronchoalveolar lavage fluid was used for estimation of NO, non-enzymatic and enzymatic Santioxidants. NO was measured using Griess reagent which measures NO indirectly as nitrite as previously described Miranda et al. [47]. Total antioxidant capacity (TAC) was measured using trolox equivalent antioxidant capacity (TEAC) assay which measures radical scavenging activity of antioxidants according to the method described by Arnao et al. [48]. The sample antioxidants reduce the blue ABTS^•+^ radical to its neutral, colourless form, ABTS (2, 2′-azino-bis-(3-ethylbenzthiazoline-6-sulphonic acid). Trolox (6-hydroxy-2, 5, 7, 8-tetramethylchroman-2-carboxylic acid) and was used for construction of the standard curve. Results were expressed as mg/mL Trolox equivalent (TE). Malondialdehyde (MDA), a product of lipid peroxidation which gives a pink-red colour with thiobarbituric acid was measured at 535 nm as described previously by Todorova et al. [49]. Reduced glutathione (GSH) levels were determined as previously reported by Owens and Belcher [50] using Ellman’s reagent (5, 5 dithiobis 2-nitrobenzoic acid) which reacts with reduced GSH resulting in a yellow colour chromophore, 5- thionitrobenzoic acid (TNB) with intensity proportionate to the GSH concentration in samples, read at 415 nm and expressed in ng/mL. Superoxide dismutase (SOD) activity was measured using a commercial assay kit (Cayman, Ann Arbor, USA) as per manufacturer’s instruction. SOD activity of the BALF samples was expressed in U/mL.

### Measurement of cytokines and OVA-specific immunoglobulin E (IgE) in serum

Commercial kits were used for measurement of serum levels of ovalbumin specific IgE antibodies (Shang Hai Korain Biotech Co. Ltd., Shanghai, China), Th2 specific cytokines IL-4 (Cloud-Clone Corp, USA), IL-5, IL-13 and Th1 specific cytokine TNF-α (Elabscience Biotechnology Co., Ltd., USA) following manufacturer’s instructions.

### Histological analysis of lung tissue

Lung tissue fixed in 10% buffered formalin was subjected to standard procedures for fixing, washing, dehydration and paraffin embedding as previously described by us [51]. Following that, 5 μm sections of the tissue were cut using a sledge microtome (Microm HM355s) and mounted on clean slides. Slides were stained with haematoxylin and counterstained with eosin for light microscopic examination. Images were taken using a digital microscope (Leica DMD108, Wetzlar, Germany). The degree of lung tissue peribronchial inflammation was scored as described previously by He et al. [52]. Using a treatment-blind reader, the score of lung inflammation was assessed according to the following criteria scale: 0, no inflammation; 1, mild inflammation; 2, moderate inflammation; 3, marked inflammation; and 4, severe inflammation. Mean scores for the treatment groups were compared.

### Data presentation and statistical analysis

Data was analysed using GraphPad Prism Version 5 statistical package and presented as mean ± standard error of the mean (SEM) in tables and figures. One-way analysis of variance (ANOVA) was used to compare means between groups followed by Tukey’s multiple-comparison test. A *p* ≤ 0.05 was considered statistically significant. Comparative qualitative description of histology sections was made.

## Results

### LC-MS analysis of phenolic compounds in *Lippia javanica*

*L. javanica* was shown to be rich in phenolic compounds, the most abundant are shown in Table 1. Among the phenolic acids identified by LC-MS, syringic acid showed the highest abundance (25.34%) in *L. javanica* then > protocatechuic acid (21%) > p-coumaric acid (16.65) > caffeic acid (14%) > vanillic acid (10.87%), the five making up 88% of the total measured phenolics in *L. javanica*. The other phenolic acids were in relatively lower abundance (Table 1).

**Table 1:** Phenolic acids identified in tea samples of *L. javanica* by LCMS

Compound	Concentration (μg/L)	Percent (%)
Trans-cinnamic acid	64.27 ± 5.3	4.87
Syringaldehyde	41.73 ± 2.9	3.16
Vanillic acid	143.38 ± 5.4	10.87
Protocatechuic acid	277.23 ± 23.0	21.00
Syringic acid	334.40 ± 24.2	25.34
p-coumaric acid	219.67 ± 11.6	16.65
Gallic acid	27.66 ± 1.3	2.10
Ferulic acid	27.23 ± 2.1	2.06
Caffeic acid	184.20 ± 8.5	13.96

Values expressed as mean ± standard error of mean, *n* = 3.

### Net body weight gain of treated animals

**Fig. 2:**
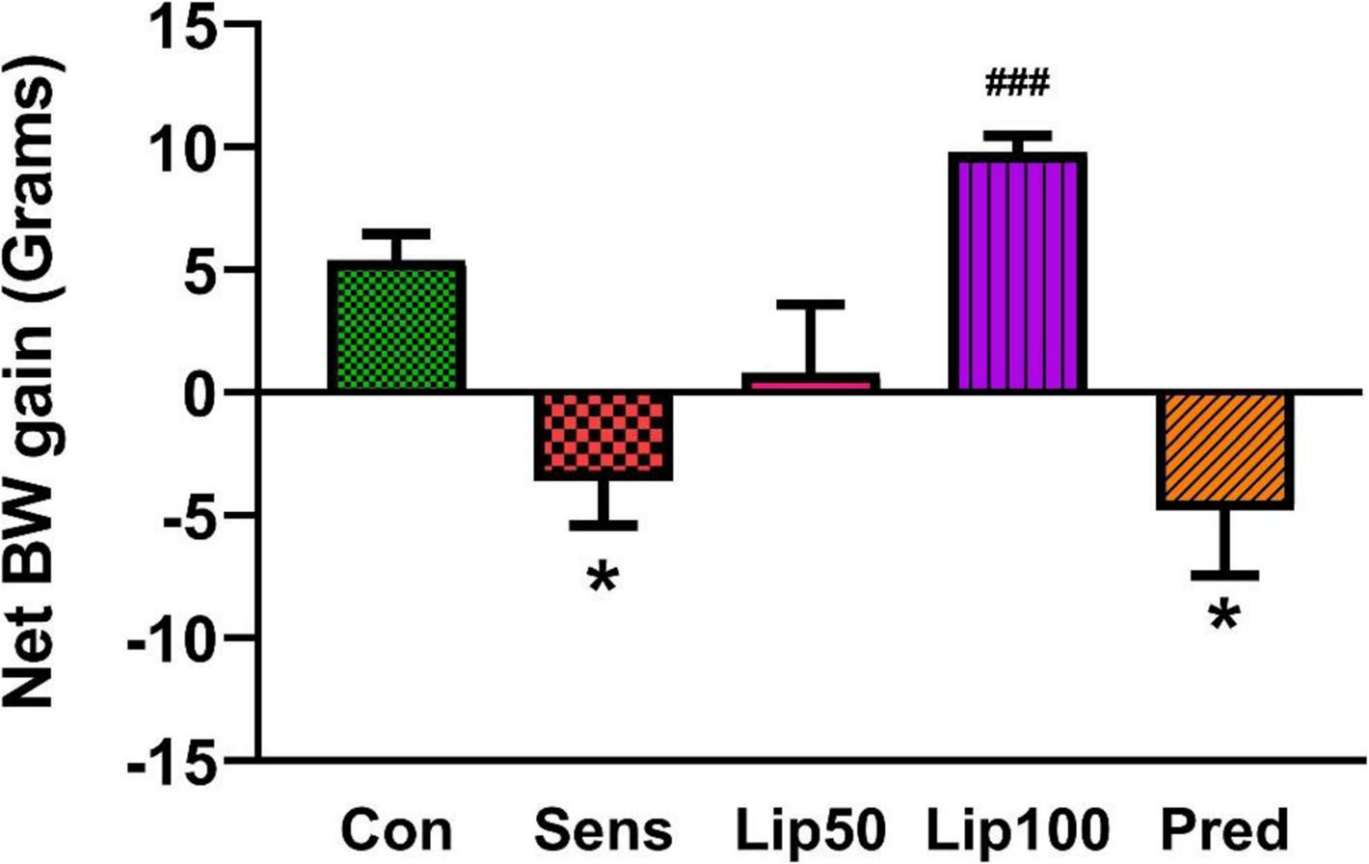
**Effect of** ***L. javanica*** **on net body weight gain in OVA-induced asthmatic rats.** Data are presented as mean ± standard error of the mean (SEM). Con = unsensitised control; Sens = ovalbumin sensitised control; Lip50 = ovalbumin sensitised + 50 mg/kg BW *L. javanica*; Lip100 = ovalbumin sensitised + 100 mg/kg BW *L. javanica*; Pred = ovalbumin sensitised + 10 mg/kg BW prednisolone. ^*^p < 0.05 compared to Con; ^###^*p* < 0.001 compared to Sens

After 10 weeks of treatment, the net body weight gain of the ovalbumin sensitised control and prednisolone treated animals was reduced (*p* < 0.05) compared to the unsensitized control. The lower dose (50 mg/kg) *L. javanica* treated animals showed similar (*p* > 0.05) net body weight gain to that of the unsensitized control. Moreover, the higher dose (100 mg/kg) *L. javanica* treated animals showed higher (*p* < 0.001) net body weight gain compared to the ovalbumin sensitised control (Fig. 2).

### Effect of *L. javanica* treatment on OVA-induced inflammatory cells in bronchoalveolar fluid (BALF)

**Fig. 3:**
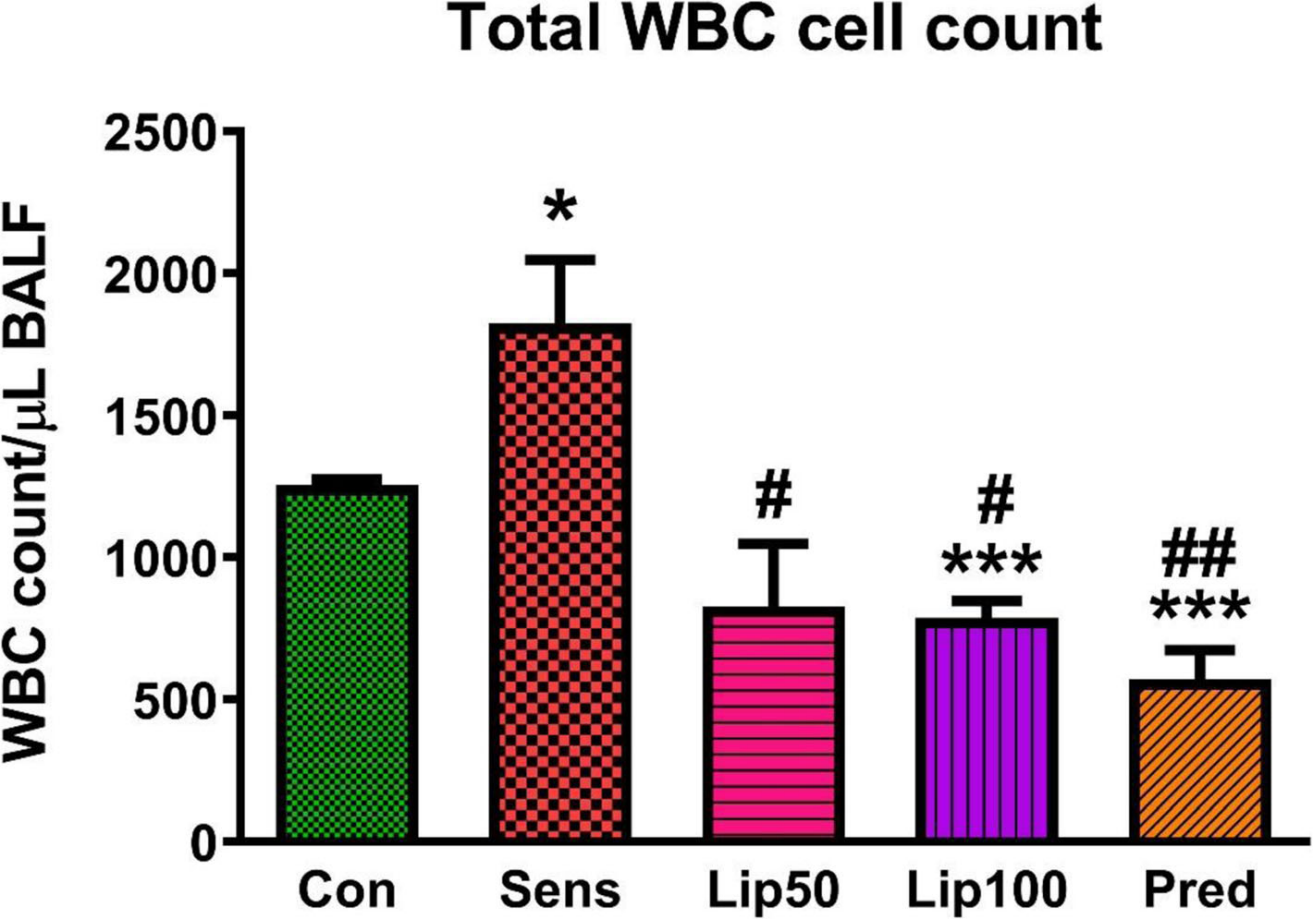
**Total white blood cell count in BALF.** Data are presented as mean ± standard error of the mean (SEM). Con = unsensitised control; Sens = ovalbumin sensitised control; Lip50 = ovalbumin sensitised + 50 mg/kg BW *L. javanica*; Lip100 = ovalbumin sensitised + 100 mg/kg BW *L. javanica*; Pred = ovalbumin sensitised + 10 mg/kg BW prednisolone. ^***^p < 0.001 compared to Con; ^#^*p* < 0.05, ^##^*p* < 0.01 compared to Sens

Overall, treatment with *L. javanica* reduced ovalbumin-induced inflammatory cell numbers in BALF. Total white blood cell count in BALF is a marker of inflammation. Exposure to ovalbumin resulted in an increase (*p* < 0.05) in total white blood count in the sensitised animals compared to the non-sensitised control. Treatment with *L. javanica* and prednisolone decreased (*p* < 0.05) total white blood cell count in all treatment groups compared to the sensitised untreated animals (Fig. 3).

**Fig. 4:**
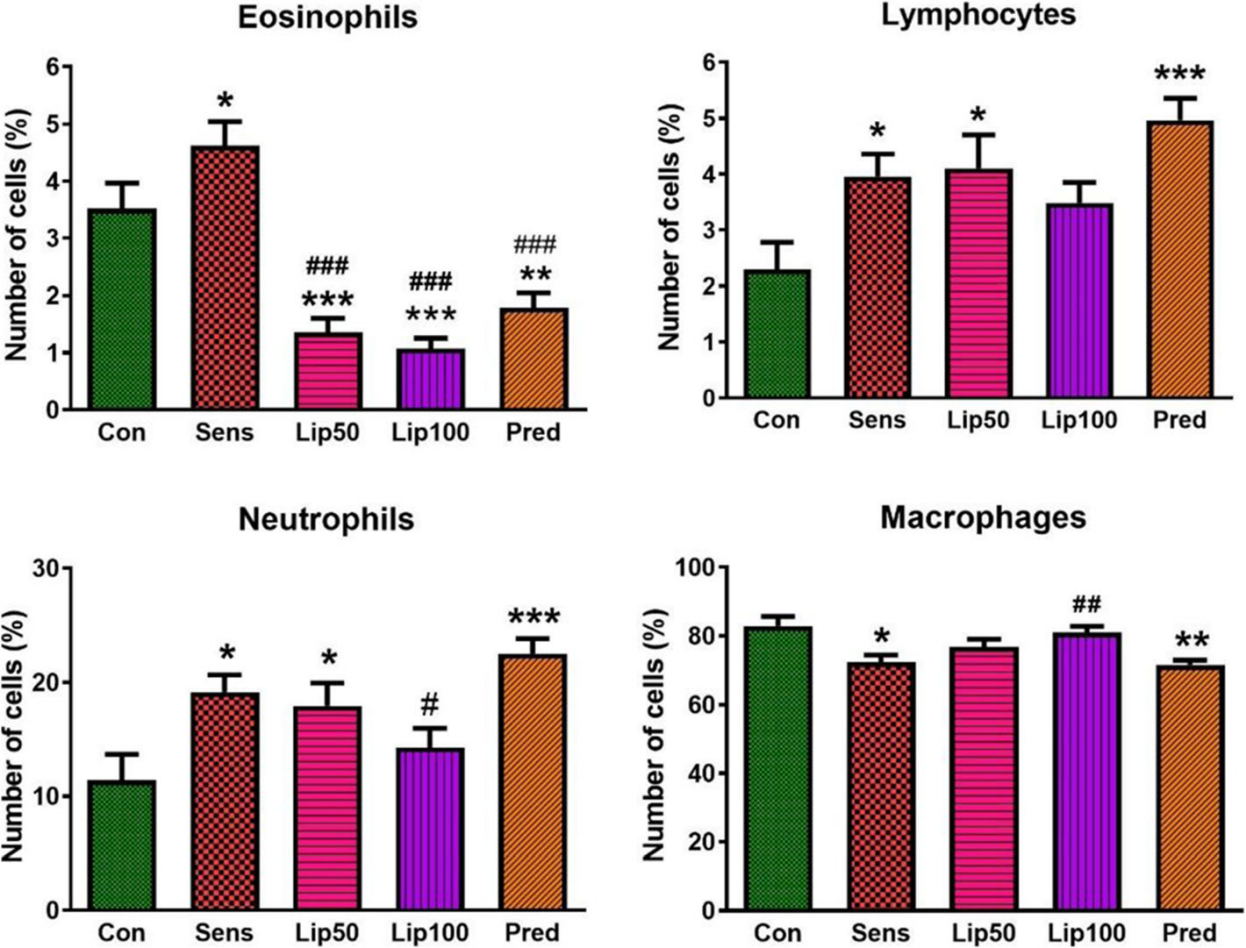
**White blood cells differential count.** Data was expressed as mean ± SEM, SEM = Standard error of the mean. Con = unsensitised control; Sens = ovalbumin sensitised control; Lip50 = ovalbumin sensitised + 50 mg/kg BW *L. javanica*; Lip100 = ovalbumin sensitised + 100 mg/kg BW *L. javanica*; Pred = ovalbumin sensitised + 10 mg/kg BW prednisolone. ^*^p < 0.05, ^**^p < 0.01, ^***^p < 0.001 compared to Con. ^#^*p* < 0.05, ^##^*p* < 0.01, ^###^*p* < 0.001 compared to Sens

Also, sensitisation with ovalbumin increased (*p* < 0.05) all inflammatory cells (neutrophils, eosinophils and lymphocytes) compared to the unsensitised control group except for macrophages which were lower. Treatment with *L. javanica* significantly (*p* < 0.001) reduced eosinophils, the specific Th2 mediated inflammatory cell compared to both unsensitised and sensitised untreated controls. Also, the higher dose of 100 mg/kg *L. javanica* lowered neutrophils compared to the sensitised untreated group. *L. javanica* treatment had no marked effect on lymphocytes and macrophages except for the higher dose of *L. javanica* (100 mg/kg) which increased macrophages when compared to the sensitised untreated group. *L. javanica* treatment had a better effect in lowering inflammatory cells compared to prednisolone, a standard anti-inflammatory drug used for asthma treatment (Fig. 4)**.**

### Effect of *L. javanica* treatment on OVA-induced oxidative stress in bronchoalveolar fluid

Nitric oxide (NO) in lung tissue contributes to nitrogen reactive species that results in lipid peroxidation. Sensitisation of rats with ovalbumin resulted in increased NO in BALF compared to the unsensitised control and treatment with *L. javanica* and prednisolone reduced (*p* < 0.05) NO in BALF compared to the sensitised control. Malondialdehyde (MDA) is a product of lipid peroxidation. Ovalbumin sensitisation increased lipid peroxidation in BALF (*p* < 0.05) compared to unsensitised control but it was reduced (*p* < 0.05) by *L. javanica* and prednisolone treatment with higher dose of *L. javanica* (100 mg/kg) showing a higher MDA lowering effect than prednisolone. Sensitisation of rats with ovalbumin reduced antioxidants; GSH and SOD but these antioxidants were increased (*p* < 0.05) in *L. javanica* treated animals compared to the sensitised control. Prednisolone treatment showed no change in GSH and SOD levels compared to the sensitised control. Equally, ovalbumin sensitisation reduced the TAC but *L. javanica* and prednisolone treatment both increased (*p* < 0.05) the TAC compared to the sensitised control (Table 2).

**Table 2:** Effect of *L. javanica* treatment on antioxidant parameter in bronchoalveolar fluid (BALF) of OVA- induced asthmatic rats

Parameters in BALF	Treatment Groups				
	Con	Sens	Lip50	Lip100	Pred
NO (μg/ml nitrite)	0.71 ± 0.06	1.49 ± 0.25*	0.73 ± 0.12^##^	0.78 ± 0.12^#^	0.57 ± 0.03^##^
MDA (μM)	3.06 ± 0.28	4.79 ± 0.29*	3.33 ± 0.55^#^	2.43 ± 0.36^##^	3.20 ± 0.29^##^
Reduced GSH (ng/ml)	59.10 ± 2.24	48.80 ± 1.59**	63.82 ± 1.52^###^	65.78 ± 2.34^###^	53.43 ± 2.07
SOD (U/ml)	9.19 ± 082	6.62 ± 0.31*	10.18 ± 0.57^##^	10.64 ± 0.64^###^	8.67 ± 0.50
TAC (mg/mL TE)	2.11 ± 0.26	1.26 ± 0.15	2.43 ± 0.24^#^	2.37 ± 0.26^#^	2.44 ± 0.23^#^

Data are presented as mean ± standard error of the mean (SEM). SEM = Standard error of the mean. Con = unsensitised control; Sens = ovalbumin sensitised control; Lip50 = ovalbumin sensitised + 50 mg/kg BW *L. javanica*; Lip100 = ovalbumin sensitised + 100 mg/kg BW *L. javanica*; Pred = ovalbumin sensitised + 10 mg/kg BW prednisolone. NO = nitric oxide; TAC = total antioxidant capacity; MDA = malondialdehyde; GSH = glutathione; SOD = superoxide dismutase. ^*^p < 0.05, ^**^p < 0.01 compared to Con; ^#^*p* < 0.05, ^##^*p* < 0.01, ^###^*p* < 0.001 compared to Sens.

### Effect of *L. javanica* treatment on OVA-induced IgE levels in serum

**Fig. 5:**
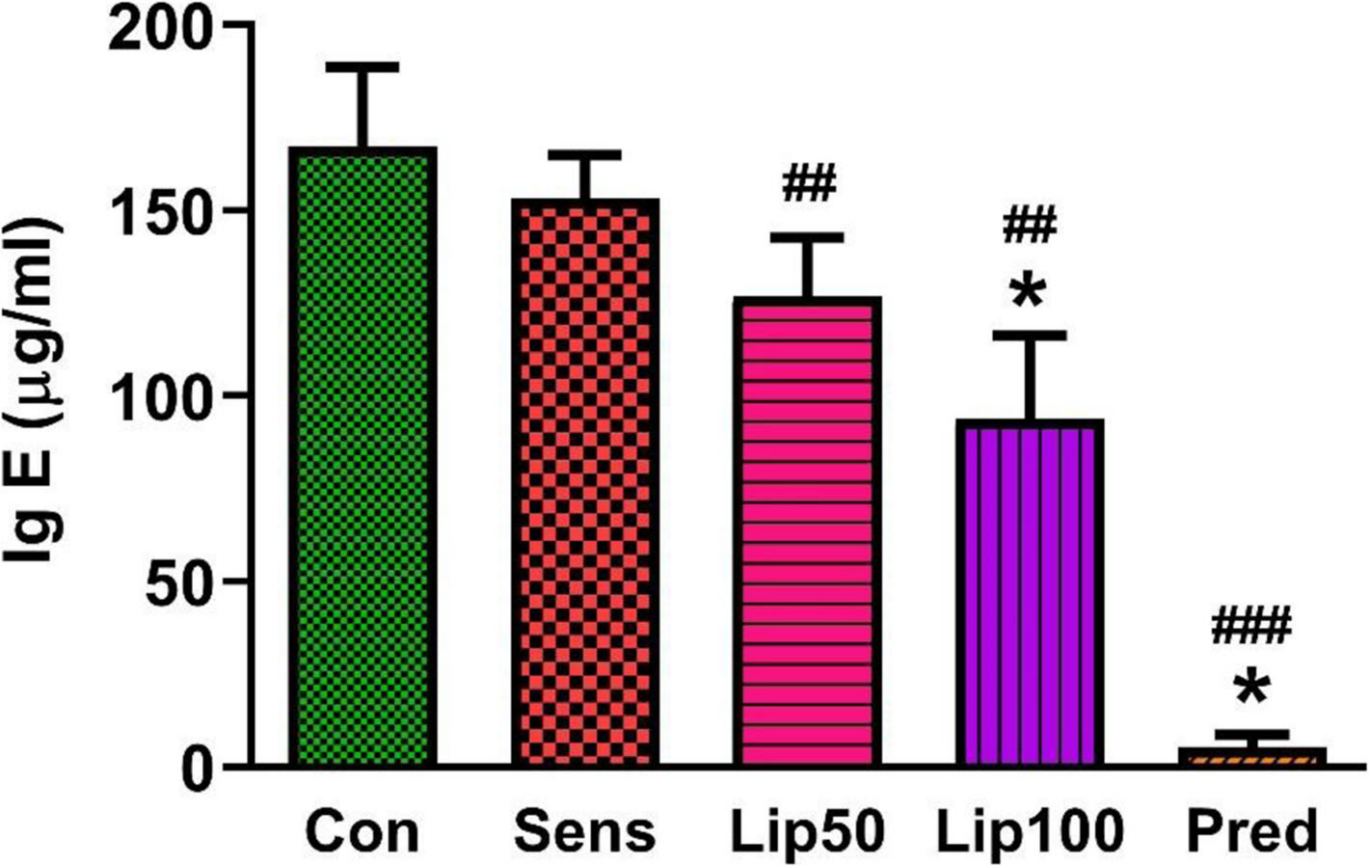
**Serum ovalbumin-specific IgE concentration.** Data was expressed as mean ± SEM. SEM = Standard error of the mean. Con = unsensitised control; Sens = ovalbumin sensitised control; Lip50 = ovalbumin sensitised + 50 mg/kg BW *L. javanica*; Lip100 = ovalbumin sensitised + 100 mg/kg BW *L. javanica*; Pred = ovalbumin sensitised + 10 mg/kg BW prednisolone. ^*^p < 0.05 compared to Con, ^##^*p* < 0.01; ^###^*p* < 0.001 compared to Sens

Ig E is an antibody associated with allergic immune response. The OVA-induced Ig E level was similar (*p* > 0.05) between the sensitised and unsensitised controls. Treatment with *L. javanica* and prednisolone reduced (*p* < 0.01) OVA-induced Ig E level compared to the sensitised group. The high dose of *L. javanica* and prednisolone treatments reduced (*p* < 0.05) OVA-induced Ig E level compared to unsensitised control with prednisolone showing the highest effect on Ig E (Fig. 5).

### Effect of *L. javanica* treatment on OVA-induced inflammatory cytokine levels in serum

**Fig. 6:**
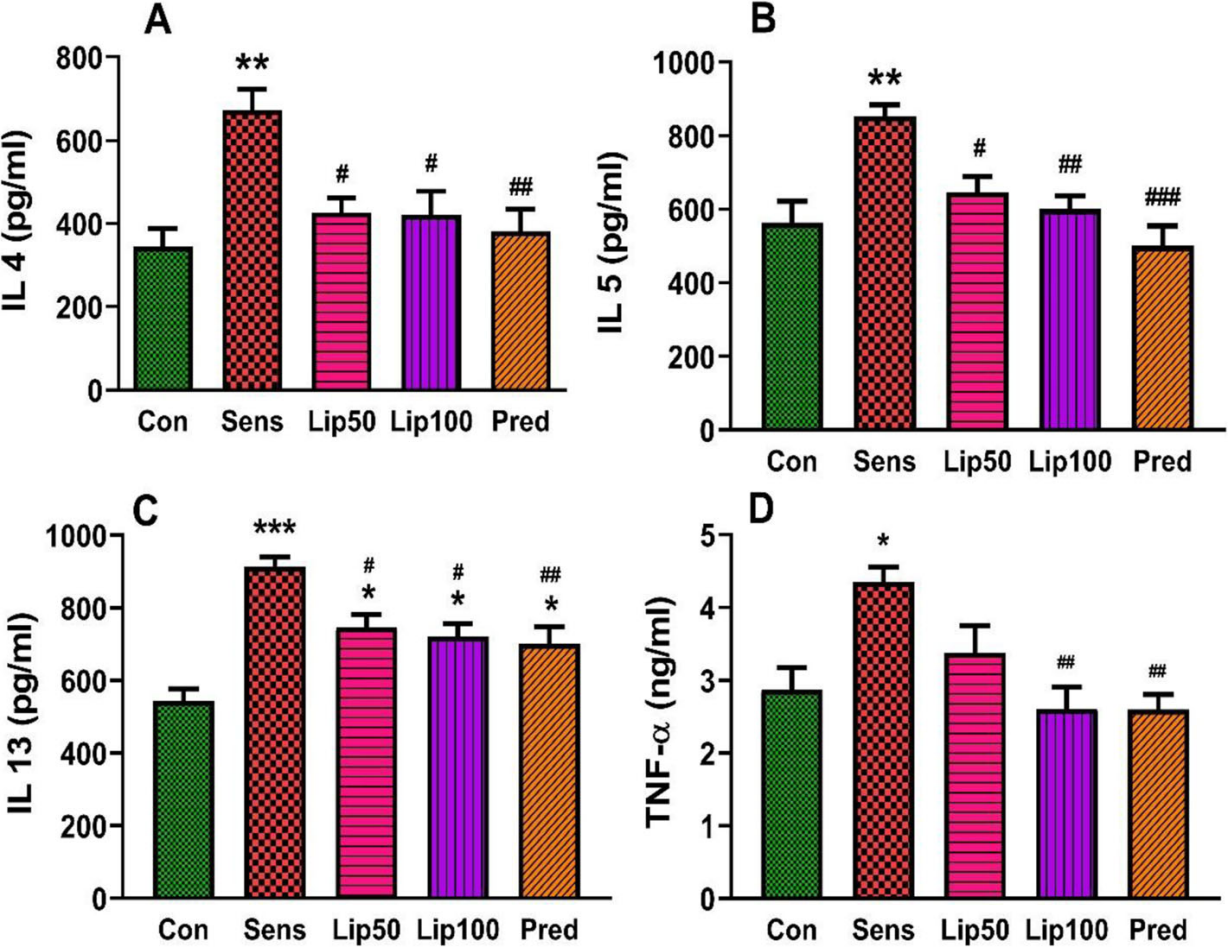
**Inflammatory cytokine concentration. Effect of** ***L. javanica*** **on serum IL-4 (A), IL-5 (B), IL-13 (C) and TNF-α (D).** Data was expressed as mean ± SEM. SEM = Standard error of the mean. Con = unsensitised control; Sens = ovalbumin sensitised control; Lip50 = ovalbumin sensitised + 50 mg/kg BW *L. javanica*; Lip100 = ovalbumin sensitised + 100 mg/kg BW *L. javanica*; Pred = ovalbumin sensitised + 10 mg/kg BW prednisolone. ^*^p < 0.05, ^**^p < 0.01, ^***^p < 0.001 compared to Con; ^#^*p* < 0.05, ^##^*p* < 0.01, ^###^*p* < 0.001 compared to Sens

IL-4, IL-5, IL-13 and TNF-α are cytokines that promote the proliferation and activation of inflammatory cells to release inflammatory molecules. Sensitisation with ovalbumin increased (*p* < 0.05) serum IL-4 (Fig. 6A), IL-5 (Fig. 6B), IL-13 (Fig. 6C) and TNF-α (Fig. 6D) compared to the unsensitised control. Treatment with *L. javanica* and prednisolone resulted in a decrease (*p* < 0.05) in these cytokines compared to the sensitised control. This reduction in cytokines by *L. javanica* and prednisolone was comparable to that of the unsensitised control for IL-4, IL-5 and TNF-α. Also, the effect of *L. javanica* treatment on cytokine reduction especially the higher dose was comparable to that of prednisolone (Fig. 6).

### Effect of *L. javanica* on OVA-induced histopathological changes in lung tissue

**Fig. 7:**
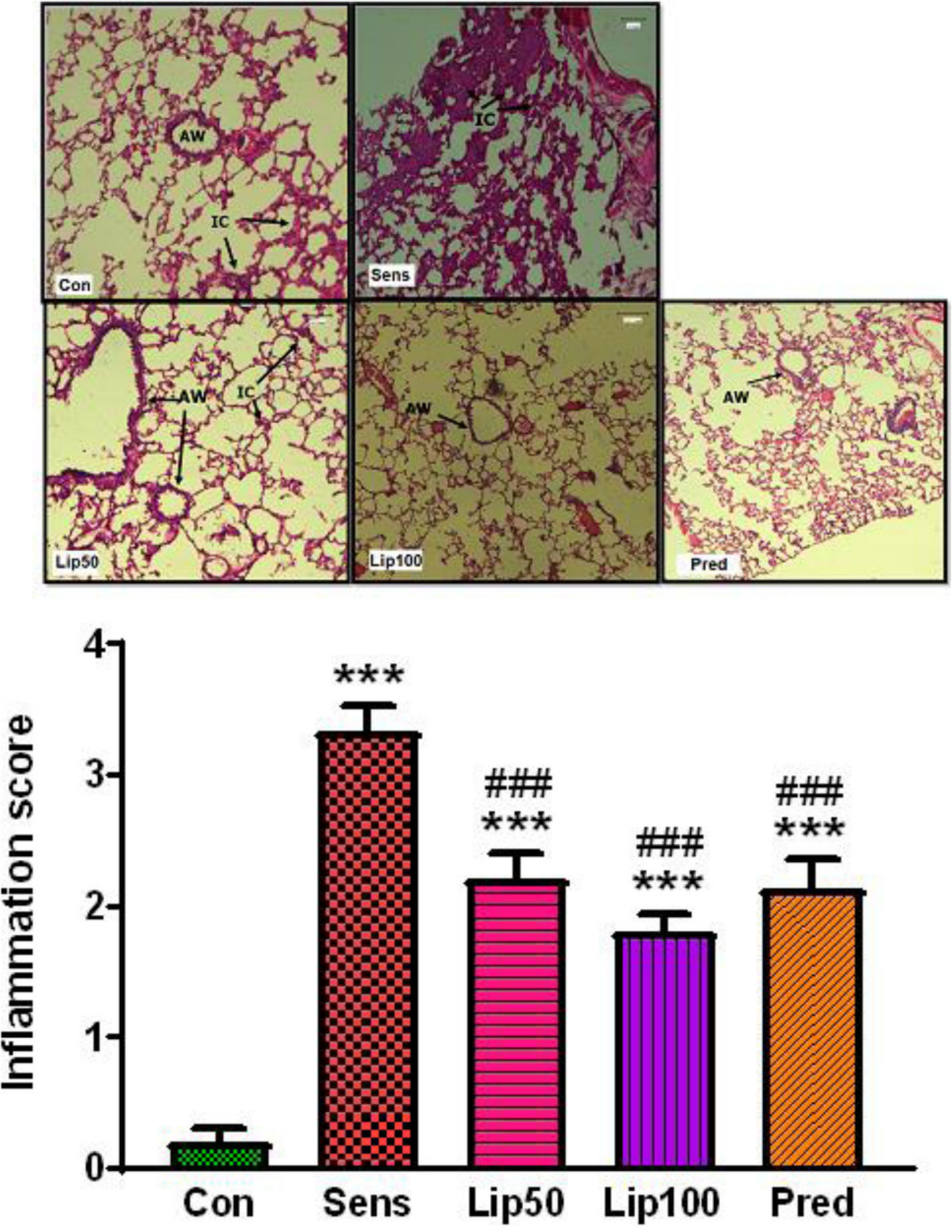
**Effect of** ***L. javanica*** **on inflammation in the lung tissue of OVA-induced asthmatic rats (H & E staining, × 20 magnification; ruler bar = 100** μm**).** IC = Inflammatory cells. AW = Airway. Con = unsensitised control; Sens = ovalbumin sensitised control; Lip50 = ovalbumin sensitised + 50 mg/kg BW *L. javanica*; Lip100 = ovalbumin sensitised + 100 mg/kg BW *L. javanica*; Pred = ovalbumin sensitised + 10 mg/kg BW prednisolone. ^***^p < 0.001 compared to Con; ^###^*p* < 0.001 compared to Sens

Ovalbumin sensitisation resulted in dense inflammatory cell infiltration into lung parenchyma and surrounding airways. Treatment with *L. javanica* resulted in a dose dependant reduction of inflammatory cells. High dose 100 mg/kg *L. javanica* and prednisolone showed no inflammatory cell invasion. Assessing the degree of lung tissue peribronchial inflammation using scores showed that ovalbumin sensitisation resulted in marked to severe inflammation which was reduced (*p* < 0.05) to moderate inflammation by *L. javanica* and prednisolone (Fig. 7).

### Effect of *L. javanica* on OVA-induced histopathological changes in *Airway smooth muscle*

**Fig. 8:**
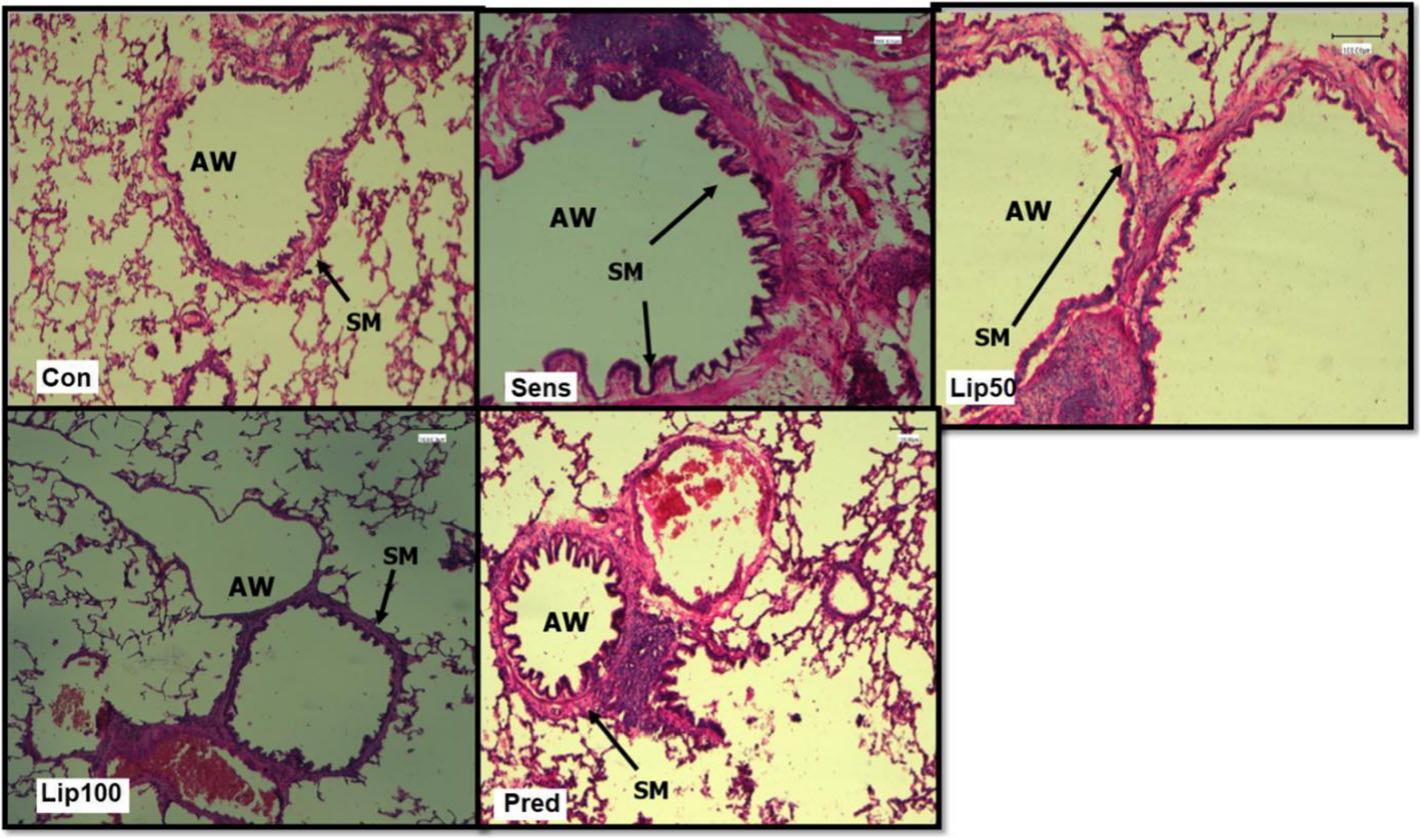
**Effect of** ***L. javanica*** **on airway smooth muscle thickness in OVA-induced asthmatic rats (H & E staining, × 20 magnification; ruler bar = 100 μm).** SM = Smooth muscle; AW = Airway. Con = unsensitised control; Sens = ovalbumin sensitised control; Lip50 = ovalbumin sensitised + 50 mg/kg BW *L. javanica*; Lip100 = ovalbumin sensitised + 100 mg/kg BW *L. javanica*; Pred = ovalbumin sensitised + 10 mg/kg BW prednisolone

Sensitisation with ovalbumin resulted in thickening of bronchiole smooth muscle with signs of lung interstitial oedema. Treatment with low dose 50 mg/kg *L. javanica* reduced smooth muscle thickening with some remnants of oedema. However, the high dose 100 mg/kg *L. javanica* and prednisolone prevented smooth muscle thickening which remained comparable to the unsensitised control (Fig. 8).

## Discussion

Asthma is a type 1 allergic reaction which is mediated by Th2 cells. Airway sensitisation to specific allergens is the initial trigger for asthma development. In this study, ovalbumin was the antigen used to trigger allergic response and airway inflammation. The preferential activation of Th2 cells by APCs lead to the production of cytokines including: IL-4, IL-5 and IL-13 [53] that results in the activation and proliferation of inflammatory cells including eosinophils, lymphocytes, neutrophils and macrophages to the site of inflammation. These cells trigger the release of inflammatory mediators including histamine, acetylcholine, leukotrienes and prostaglandins leading to inflammation [54]. To assess inflammatory cell migration, we performed total and differential white blood cell counts in BALF. OVA-sensitized animals had significant increase in total white blood cell count (WBC) as well as eosinophils, lymphocytes and neutrophils in BALF compared to the unsensitised animals. Thus, OVA-sensitisation increased inflammatory cells. This confirmed that inflammatory process was active in the lungs of OVA-sensitized animals. *L. javanica* treated animals showed reduced WBC as well as lymphocytes, eosinophils, and neutrophils compared to the OVA-sensitised untreated animals with eosinophils showing a mark reduction. The higher dose of *L. javanica* was more effective in reducing these cells. Thus, treatment with *L. javanica* resulted in a dose dependant reduction of inflammatory cells. This finding suggests that *L. javanica* prevented inflammatory cell migration especially eosinophils at the site of inflammation. Inhibition of inflammatory cell migration is a known mode of action by corticosteroids to decrease airway hyper-responsiveness and inflammatory response to allergens by down regulating eosinophil and mast cell activation [55]. Thus, *L. javanica* may possess similar therapeutic effect as corticosteroids. Moreover, prednisolone, a known corticosteroid which is known to lower lymphocytes at high doses [55] showed no effect in lowering lymphocytes in this study. Hence, the dose of pregnisolone used was tolerated by the animals and thus suitable for the study.

Inflammatory cells are recruited at inflammatory sites by cytokines including interleukin (IL)-4, IL-5, IL-13 and TNF-α following Th2 cells activation. IL-4 is known to activate memory B cells to secrete immunoglobulins and to induce class switching of IgM to IgE, thereby incorporating IgE on mast cells. Also, IL-13 is known to stimulate the activation, differentiation, and proliferation of B cells into plasma cells and facilitate class switching from immunoglobulin (Ig) G to Ig E [13]. The cross-linkage of IgE molecule on mast cells with an allergen causes its degranulation and release of histamine, leukotrienes, and other mediators that exacerbates asthma [56]. OVA-induced IgE was reduced in *L. javanica* treated animals compared to OVA-sensitised untreated animals and this corresponded to a reduction in IL-4 and IL-13 in *L. javanica* treated animals and the effect was dose dependent. These findings suggest that *L. javanica* prevented IgE production and limited mast cell priming by preventing the release of IL-4 and IL-13. IL-13 is also involved in tissue remodelling by stimulating fibroblasts to synthesize collagen and it induces the production of mucus by promoting the differentiation, proliferation and secretory function of lung epithelial goblet cells [57]. IL-13 as well as IL-5 and TNF-α are involved in the activation of macrophages and migration of inflammatory cells; eosinophils, neutrophils and macrophages to inflammatory cells [10]. Treatment with *L. javanica* resulted in a decrease in these inflammatory cytokines which corresponds to the reduction of inflammatory cells in BALF. Considering that infiltration of inflammatory cells, increased Ig E and mucus secretion are mediated by Th2 cells resulting in hyper-responsiveness of the airways [58], *L. javanica* has shown to inhibit Th2 cell activation thereby preventing hyper-responsiveness and airway inflammation.

Inflammatory cell invasion of tissues is associated with increased oxidative stress as activated eosinophils, monocytes, neutrophils, and macrophages have shown to generate reactive oxygen species (ROS) [59]. It has been reported that elevated levels of reactive oxygen species (ROS) can triggers some intracellular signalling cascades such as MAPK and NF-κB that are potentially proinflammatory leading to the expression of inflammatory cytokines and chemokines [26]. In the lungs, nitric oxide (NO) which is the main nitrogen species can further react with H_2_O_2_ and O_2_^−^ to form more reactive nitrogen species (RNS) such as peroxynitrile (ONOO^−^) which can damage lung tissue [60]. In the airways, lipid peroxidation, a marker for oxidative damage is associated with increased airway hyper-responsiveness in asthma as excess production of ROS correlates with the degree of airway hyper-responsiveness [27]. Oxidative stress in the airways enhances and progresses the symptoms of the existing airway inflammation, elevate hyper-responsiveness of the bronchioles, increase mucus secretion, and increase pro-inflammatory chemicals. The antioxidant defense system is eminent for the prevention of oxidative stress. Antioxidant deficiency has been observed in asthma as a marked reduction in plasma antioxidant capacity during exacerbations in asthmatic patients while CuZn-SOD activity was found to be lower in asthmatics than in normal subjects [61]. Moreover, reduced levels of glutathione has been observed in BALF of asthmatic patients [62]. OVA-sensitized animals showed marked increased NO and MDA formation with reduced level of GSH, SOD and total antioxidant capacity. Treatment with *L. javanica* decreased lung NO levels and lipid peroxidation while a remarkable increased in SOD, GSH and TAC level were observed in a dose-dependent manner. Previous in vitro studies have shown *L. javanica* to possess free radical and NO scavenging as well as potent antioxidant properties [63, 64].

Asthmatic lung tissue is often characterised by increased inflammatory cells, eosinophilia, increased mucosal goblet cells, bronchial smooth muscle hypertrophy, thickened basement membrane, and airway wall oedema [20]. These histological features were evident in the lung airway tissue and muscle of OVA-sensitised untreated animals as ovalbumin sensitisation resulted in dense inflammatory cell infiltration into lung parenchyma and surrounding airways. Treatment with *L. javanica* reduced inflammatory cells infiltration in the tissues, smooth muscle thickening, oedema, bronchial smooth muscle hypertrophy and mucosal goblet cells. Generally, the effect of *L. javanica* in suppressing inflammation, reducing cytokines and inflammatory cell infiltration was comparable to that of prednisolone, an oral corticosteroid which is a known treatment against asthma. Moreover, *L. javanica was* more efficient in preventing oxidative stress and improving the antioxidant defence in ova-sensitised animals than prednisolone*.* Though prednisolone is a known drug used to treat the exacerbation of asthma, it was shown to reduce the body weight of animals, one of the side effects of prednisolone. Corticosteroids are known to cause muscle atrophy and decrease in bone density ultimately contributing to total weight loss [65]. Phytochemical profiling by LC-MS showed *L. javanica* to be rich in phenolic acids of which syringic acid, protocatechuic acid, p-coumaric acid, caffeic acid and vanillic acid contributed 88% of the total phenolics measured in *L. javanica* while trans-cinnanic acid, syringaldehyde, gallic acid and ferulic were in lower concentrations. The five abundant phenolics have been shown experimentally to possess antioxidant and anti-inflammatory effects [66–71]. It is therefore reasonable to associate these phenolic compounds to the observed effects observed in *L. javanica* treated rats. *L. javanica* has previously been shown to contain bioactive compounds such as polyphenols, flavonoids, flavonols and proanthocyanidin [37, 63]. Flavonoids and phenols have been shown to possess anti-inflammatory, bronchodilation and anti-asthmatic effects by inhibiting the synthesis of Th2 type cytokines and release of chemical mediators [64]. Therefore, the possible mechanisms for the anti-inflammatory and antioxidant effects of *L. javanica* may be through these phenolic compounds which in concert results in the suppression of Th2 cell mediated immune responses and the associated immune cell-induced oxidative stress culminating in the observed anti-asthmatic effects.

Though this study showed *L. javanica* to supress Th2 cell activation in ovalbumin-sensitized rats thereby attenuating airway inflammation, it was limited in that it did not assess the role of *L. javanica* on T-cell subsets which have recently been shown to be involved in the pathogenesis of airway inflammation in asthma [72]. Also, airway resistance and lung compliance; aspects of lung mechanics which are other indicators of allergic asthma [73] were not assessed. More so, the estrous cycle in females which may modulate the inflammatory and oxidative stress responses [74] was not controlled in this study. However, the study design was suitable to induce allergic asthma and the biochemical assessments via immunologic, inflammatory, and oxidative stress indicators has provided sufficient preliminary data on the effect of *L. javanica* in the suppression of airway inflammation in ova-induced asthma. Thus, further studies will be needed to address these limitations and ascertain this finding.

## Conclusion

*L. javanica* was effective in suppressing inflammatory cell infiltration and their cytokines, and decreased inflammation-induced oxidative stress in ovalbumin-sensitized rats. These effects may be attributed to the diverse array of phenolic acid content in *L. javanica* leaves. This study showed that ingestion of an equivalent of one or two cups of *L. javanica* tea per day has significant anti-inflammatory, antioxidant and anti-asthmatic properties. Indeed, *L. javanica* tea infusion has potential for use in the treatment and/or prophylaxis for asthma and other infective and non-infective airway inflammatory ailments as used traditionally by various communities. Therefore, this study validates the traditional use of *L. javanica* in the treatment of respiratory disorders and suggests that *L. javanica* reduces allergic airway inflammation by the suppression of oxidative stress and Th2-mediated immune response.

## Data Availability

All data generated or analysed during this study are included in this published article.

## Abbreviations

Th: T-helper lymphocytes
IL: interleukin
TNF-α: Tumour necrosis factor alpha
PGs: prostaglandins
iNOS: inducible nitric oxide synthase
SOD: Superoxide dismutase
GSH: Glutathione
ROS: Reactive oxygen species
QTOF: Quadrupole time-of-flight
MS: Mass spectrometer
UPLC: Ultra-performance liquid chromatography
OVA: ovalbumin
BALF: Bronchoalveolar lavage fluid
NO: Nitric oxide
TAC: Total antioxidant capacity
MAPK: Mitogen-activated protein kinase
NF-κB: nuclear factor-κB
MDA: Malondialdehyde
APCs: Antigen presenting cells
WBC: White blood cell
NO_2_: Nitrite
RNS: Reactive nitrogen species
H_2_O_2_: Hydrogen peroxide
O^−^_2_: Superoxide
ONOO^−^: Peroxynitrile
LC-MS: Liquid chromatography- mass spectrometer

## References

[1] Global Asthma Network. The Global Asthma Report 2018. Auckland, New Zealand: Global Asthma Network, 2018. Accessed 12th July, 2020. http://www.globalasthmareport.org/resources/global_asthma_report_2018.pdf

[2] Murphy DM, O'Byrne PM (2010). Recent advances in the pathophysiology of asthma. Chest.

[3] Mims JW (2015). Asthma: definitions and pathophysiology. Int Forum Allergy & Rhinol.

[4] Romanet-Manent S, Charpin D, Magnan A, Lanteaume A, Vervloet D (2002). EGEA cooperative group. Allergic vs nonallergic asthma: what makes the difference?. Allergy.

[5] GBD 2016 Disease and Injury Incidence and Prevalence Collaborators. Global, regional, and national incidence, prevalence, and years lived with disability for 328 diseases and injuries for 195 countries, 1990–2016: a systematic analysis for the Global Burden of Disease Study 2016. Lancet. 2017 Sep 16; 390 (10100):1211–1259. doi: 10.1016/S0140-6736(17)32154-2. Erratum in: Lancet. 2017; 28; 390 (10106): e38. 10.1016/S0140-6736(17)32154-2 PMC5605509 28919117

[6] Bousquet J, Clark TJ, Hurd S, Khaltaev N, Lenfant C, O'byrne P, Sheffer A (2007). GINA guidelines on asthma and beyond. Allergy.

[7] Adeloye D, Chan KY, Rudan I, Campbell H (2013). An estimate of asthma prevalence in Africa: a systematic analysis. Croat Med J.

[8] Ait-Khaled N, Pearce N, Anderson HR (2009). Global map of the prevalences of symptomsof rhinoconjuctivitis on children in Africa: the international study of asthma and allergies in Africa. Allergy.

[9] Jakubzick C, Tacke F, Llodra J, van Rooijen N, Randolph GJ (2006). Modulation of dendritic cell trafficking to and from the airways. J Immunol.

[10] Kim H, Mazza J (2011). Asthma. Allergy, Asthma Clin Immunol.

[11] Chedevergne F, Bourgeois M, Blic J, Scheinmann P. The role of inflammation in childhood asthma. Arch Dis Child 2000; ^**82**^(II):ii6–ii9. 10.1136/adc.82.suppl_2.ii6 PMC1765084 10833470

[12] Murdoch JR, Lloyd CM (2010). Chronic inflammation and asthma. Mutation Res.

[13] Gould HJ, Sutton BJ (2008). IgE in allergy and asthma today. Nat Rev Immunol..

[14] Pope SM, Brandt EB, Mishra A, Hogan SP, Zimmermann N, Matthaei KI, Foster PS, Rothenberg ME (2001). IL-13 induces eosinophil recruitment into the lung by an IL-5–and eotaxin-dependent mechanism. J Allergy Clin Immunol.

[15] Menzies-Gow AN, Flood-Page PT, Robinson DS, Kay AB (2007). Effect of inhaled interleukin-5 on eosinophil progenitors in the bronchi and bone marrow of asthmatic and non-asthmatic volunteers. Clin Exp Allergy.

[16] Chibana K, Trudeau JB, Mustovitch AT, Hu H, Zhao J, Balzar S, Chu HW, Wenzel SE (2008). IL-13 induced increases in nitrite levels are primarily driven by increases in inducible nitric oxide synthase as compared with effects on arginases in human primary bronchial epithelial cells. Clin Exp Allergy.

[17] Kasaian MT, Miller DK (2008). IL-13 as a therapeutic target for respiratory disease. Biochem Pharmacol.

[18] Brightling C, Berry M, Amrani Y (2008). Targeting TNF-alpha: a novel therapeutic approach for asthma. J Allergy Clin Immunol.

[19] Fernhoff NB, Derbyshire ER, Marletta MA (2009). A nitric oxide/cysteine interaction mediates the activation of soluble guanylate cyclase. Proc Nat Aca Sci USA.

[20] Shankar PS (2017). Airway pathology in bronchial asthma. J Med Sci.

[21] Urbankowski O, Przybyłowski T (2016). Methods of airway resistance assessment. Pneumonol Alergol Pol.

[22] Arora P, Ansari SH, Anjum V, Mathur R, Ahmad S (2017). Investigation of anti-asthmatic potential of Kanakasava in ovalbumin-induced bronchial asthma and airway inflammation in rats. J Ethnopharmacol.

[23] Mukherjee AA, Kandhare AD, Rojatkar SR, Bodhankar SL (2017). Ameliorative effects of Artemisia pallens in a murine model of ovalbumin-induced allergic asthma via modulation of biochemical perturbations. Biomed Pharmacother.

[24] Warren KJ, Dickinson JD, Nelson AJ, Wyatt TA, Romberger DJ, Poole JA (2019). Ovalbumin-sensitized mice have altered airway inflammation to agriculture organic dust. Respir Res.

[25] Riedl MA, Nel AE (2008). Importance of oxidative stress in the pathogenesis and treatment of asthma. Curr Opinion allergy Clin Immunol.

[26] Cho YS, Moon H-B (2010). The role of oxidative stress in the pathogenesis of asthma. Allergy Asthma Immunol Res.

[27] Sahiner UM, Birben E, Erzurum S, Sackesen C, Kalayci O (2011). Oxidative stress in asthma. World Allergy Organization (WAO) J.

[28] Andrianjafimasy M, Zerimech F, Akiki Z, Huyvaert H, Le Moual N, Siroux V, Matran R, Dumas O (2017). Nadif R. Eur Respir J.

[29] Aldakheel FM, Thomas PS, Bourke JE, Matheson MC, Dharmage SC, Lowe AJ (2016). Relationships between adult asthma and oxidative stress markers and pH in exhaled breath condensate: a systematic review. Allergy.

[30] Omenaas E, Fluge Ø, Buist AS, Vollmer WM, Gulsvik A. Dietary vitamin C intake is inversely related to cough and wheeze in young smokers. Respiratory Med. 2003;97(2): :134–142. 10.1053/rmed.2003.1439 12587963

[31] Comhair SA, Erzurum SC (2010). Redox control of asthma: molecular mechanisms and therapeutic opportunities. Antioxid Redox Signal.

[32] Tripathi KD (2013). Essentials of medical pharmacology. New Delhi: Jaypee Brothers Medical Publishers Ltd.

[33] Kling S, Zar HJ, Levin ME, Green RJ, Jeena PM, Risenga SM, Thula SA, Goussard P, Gie RP (2013). Guideline for the management of acute asthma in children: 2013 update. S Afr Med J.

[34] York T, De Wet H, Van Vuuren SF. Plants used for treating respiratory infections inrural Maputaland,KwaZulu-Natal,SouthAfrica J Ethnopharmacol 2011;135:696–710, Plants used for treating respiratory infections in rural Maputaland, KwaZulu-Natal, South Africa, 3, DOI: 10.1016/j.jep.2011.03.072. 10.1016/j.jep.2011.03.072 21497646

[35] Maroyi, A., 2017. Lippia javanica (Burm. F.) Spreng.: traditional and commercial uses and phytochemical and pharmacological significance in the african and indian subcontinent. Evidence-based complementary and alternative medicine, 2017. 10.1155/2017/6746071 PMC5237467 28115974

[36] Matthew M, Chingono F, Mangezi S, Mare A, Mbazangi S. Hidden variables to Covid 19: Zimbabwe. Cambridge Open Engage. 2020. doi:10.33774/coe-2020-1mqnz

[37] Shikanga EA, Combrinck S, Regnier T (2010). South African Lippia herbal infusions: Total phenolic content, antioxidant and antibacterial activities. S Afr J Bot.

[38] Zhang H, Tsao R (2016). Dietary polyphenols, oxidative stress and antioxidant and anti-inflammatory effects. Curr Opinion Food Sci.

[39] Suleman Z (2015). Comparing the antioxidant properties of Lippia javanica with *Aspalathus linearis* (rooibos).

[40] Rautenbach M, Vlok NM, Eyéghé-Bickong HA, van der Merwe MJ, Stander MA. An electrospray ionization mass spectrometry study on the “In Vacuo” hetero-oligomers formed by the antimicrobial peptides, surfactin and gramicidin S J Am Soc Mass Spectrometry 2017; 28(8):1623–1637. 10.1007/s13361-017-1685-0 28560564

[41] Joubert E, Gelderblom WCA, Louw A (2008). deBeer D. south African herbal teas: Aspalathus linearis, Cyclopia spp. and Athrixia pylicoides- a review. J Ethnopharmacol.

[42] Percie du Sert N, Ahluwalia A, Alam S, Avey MT, Baker M, Browne WJ, Clark A, Cuthill IC, Dirnagl U, Emerson M. Reporting animal research: Explanation and elaboration for the ARRIVE guidelines 2.0. PLoS Biol 2020; 18(7): e3000411. 10.1371/journal.pbio.3000411 PMC7360025 32663221

[43] SANS. The care and use of animals for scientific purposes. South African National Standard (SANS) 2008, SANS10386: 2008.

[44] Chapman RW, Jones H, Richard J, Celly C, Prelusky D, Ting P, Hunter JC, Lamca J, Phillips JE, House A (2007). Anti-asmatic potential of chrysinon ovalbumin-induced bronchoalveolar hyperresponsiveness in rats. Eur J Pharmacol.

[45] Reagan-Shaw S, Nihal M, Ahmad N (2008). Dose translation from animal to human studies revisited. FASEB J.

[46] Walker HK, Hall WD, Hurst JW. (eds). Clinical methods: the history, physical, and laboratory examinations. Chapter 153 - The White Blood Cell and Differential Count Butterworth-Heinemann. 1990. 21250045

[47] Miranda KM, Espey MG, Wink DA (2001). A rapid, simple spectrophotometric method for simultaneous detection of nitrate and nitrite. Nitric Oxide.

[48] Arnao MB, Cano A, Acosta M (2001). The hydrophilic and lipophilic contribution to total antioxidant activity. Food Chem.

[49] Todorova I, Simeonova G, Kyuchukova G, Dinev D, Gadjeva V (2005). Reference values of oxidative stress parameters (MDA, SOD, CAT) in dogs and cats. Comparative Clin Pathol.

[50] Owens CWI, Belcher RV (1965). A colorimetric micro-method for the determination of glutathione. Biochem J.

[51] Tiya S, Sewani-Rusike CR, Shauli M (2017). Effects of treatment with Hypoxis hemerocallidea extract on sexual behaviour and reproductive parameters in male rats. Andrologia.

[52] He J, Lv L, Wang Z, Huo C, Zheng Z, Yin B, Jiang P, Yang Y, Li J, Gao Y, Xue J (2017). Pulvis Fellis Suis extract attenuates ovalbumin-induced airway inflammation in murine model of asthma. J Ethnopharmacol.

[53] Makoto K, Ishigatsubo Y, Ichiro A (2013). Pathology of asthma. Fontiers Microbiol.

[54] Barnes BJ (2008). The cytokine network in asthma and chronic obstructive pulmonary disease. J Clin Investigation.

[55] So JY, Mamary AJ, Shenoy K (2018). Asthma: diagnosis and treatment. Eur Med J.

[56] Bradding P (2003). The role of the mast cell in asthma: a reassessment. Curr Opinion in Allergy Clin Immunol.

[57] Holgate ST, Polosa R (2008). Treatment strategies for allergy and asthma. Nat Rev Immunol..

[58] Bush A, Kleinert S, Pavord ID (2015). The asthmas in 2015 and beyond: a lancet commission. Lancet.

[59] Sahiner UM, Birben E, Erzurum S, Sackesen C, Kalayci Ö (2018). Oxidative stress in asthma: part of the puzzle. Paediatric Arllergy Immunol.

[60] Mittal M, Siddiqui MR, Tran K, Reddy SP, Malik AB (2014). Reactive oxygen species in inflammation and tissue injury. Antioxidants Redox Signaling.

[61] Neil L, Misso A, Thompson PJ (2005). Oxidative stress and antioxidant deficiencies in asthma: potential modification by diet. Redox Rep.

[62] Fitzpatrick AM, Jones DP, Brown LA. Glutathione redox control of asthma: from molecular mechanisms to therapeutic opportunities. Antioxid Redox Signal 2012;15;17(2):375–408. 10.1089/ars.2011.4198 PMC3353819 22304503

[63] Asowata-Ayodele AM, Otunola GA, Afolayan AJ (2016). Assessment of the polyphenolic content, free radical scavenging, anti-inflammatory, and antimicrobial activities of acetone and aqueous extracts of Lippia javanica (Burm.F.) spreng. Pharmacog Mag.

[64] Endris A, Asfaw N, Bisrat D (2016). Chemical composition, antimicrobial and antioxidant activities of the essential oil of Lippia javanica leaves from Ethiopia. J Essential Oil Res.

[65] Ramamoorthy S, Cidlowski JA (2016). Corticosteroids-mechanisms of action in health and disease. Rheumatic Dis Clin North America.

[66] Srinivasulu C, Ramgopal M, Ramanjaneyulu G, Anuradha CM, Kumar CS (2018). Syringic acid (SA)–a review of its occurrence, biosynthesis, pharmacological and industrial importance. Biomed Pharmacother.

[67] Pei K, Ou J, Huang J, Ou S (2016). P-Coumaric acid and its conjugates: dietary sources, pharmacokinetic properties and biological activities. J Sci Food Agriculture.

[68] Pragasam SJ, Venkatesan V, Rasool M (2013). Immunomodulatory and anti-inflammatory effect of p-coumaric acid, a common dietary polyphenol on experimental inflammation in rats. Inflammation.

[69] Paciello F, Di Pino A, Rolesi R, Troiani D, Paludetti G, Grassi C, Fetoni AR (2020). Anti-oxidant and anti-inflammatory effects of caffeic acid: in vivo evidences in a model of noise-induced hearing loss. Food ChemToxicol.

[70] Calixto-Campos C, Carvalho TT, Hohmann MS, Pinho-Ribeiro FA, Fattori V, Manchope MF, Zarpelon AC, Baracat MM, Georgetti SR, Casagrande et al. Vanillic acid inhibits inflammatory pain by inhibiting neutrophil recruitment, oxidative stress, cytokine production, and NFκB activation in mice. 2015; J Natural Products, 78(8): 1799-1808. 10.1021/acs.jnatprod.5b00246 26192250

[71] Tanaka T, Takahashi R (2013). Flavonoids and asthma. Nutrients.

[72] Topalovic M, Derom E, Osadnik CR, Troosters T, Decramer M, Janssens W (2015). Airways resistance and specific conductance for the diagnosis of obstructive airways diseases. Respir Res.

[73] Lloyd CM, Hessel EM (2010). Functions of T cells in asthma: more than just T(H)2 cells. Nat Rev Immunol.

[74] Arakawa K, Arakawa H, Hueston C, M, Deak T. Effects of the Estrous Cycle and Ovarian Hormones on Central Expression of Interleukin-1 Evoked by Stress in Female Rats. Neuroendocrinol. 2014; 100: 162–177. 10.1159/000368606 25300872

